# Cephalometric wits appraisal in cleft and non-cleft individuals using AI

**DOI:** 10.6026/973206300223023

**Published:** 2026-05-31

**Authors:** Mohammad Khursheed Alam, Ahmed Ali Alfawzan

**Affiliations:** 1 Department of Orthodontics, College of Dentistry, Jouf University, Saudi Arabia; 2 Department of Dental Research Cell, Saveetha Institute of Medical and Technical Sciences, Saveetha Dental College and Hospitals, 72345 Chennai, India; 3 Department of Public Health, Faculty of Allied Health Sciences, Daffodil lnternational University, Dhaka 1216, Bangladesh; 4 Department of Orthodontics and Pediatric Dentistry, College of Dentistry, Qassim University, Saudi Arabia

**Keywords:** Cleft lip and palate (CLP), wits appraisal, Saudi, children

## Abstract

Variations in craniofacial morphology between individuals with cleft lip and palate and non-cleft populations require accurate evaluation for effective treatment planning. Therefore, it is of interest to compare demographic characteristics and Wits appraisal scores between cleft lip and palate and non-cleft individuals, while also assessing gender differences. Hence, a total of 123 participants were analyzed using descriptive statistics, independent sample tests, and ANOVA to evaluate differences across groups. The results showed no significant gender differences in Wits scores, but significant variations were observed between cleft and non-cleft groups and among different cleft types (p < 0.05). Thus, we show the importance of considering cleft-related morphological variations for individualized orthodontic and craniofacial treatment planning.

## Background:

Artificial intelligence (AI) has rapidly emerged as an important technological advancement with applications across various fields, including healthcare and dentistry. In orthodontics and cleft lip and/or palate (CLP) management, AI-based systems have been increasingly utilized for cephalometric landmark identification, diagnosis, treatment planning, and prediction of future surgical requirements. Deep learning models such as neural networks, decision trees, and random forest algorithms have demonstrated accuracy comparable to expert clinicians in identifying cephalometric landmarks and assisting in craniofacial assessment [1, 2, 3, 4]. CLP is among the most common congenital craniofacial anomalies and is associated with multiple dental and skeletal abnormalities that affect facial aesthetics and maxillary growth. Its etiology is multifactorial, involving genetic predisposition and environmental influences such as maternal smoking, alcohol use, infections, stress, and teratogenic exposure during pregnancy [5, 6, 7]. Patients with CLP frequently exhibit altered facial morphology, lip deformities, nasolabial angle discrepancies, delayed dental development, tooth agenesis, and maxillary deficiencies, all of which require multidisciplinary management [8]. Advances in imaging techniques such as cone-beam computed tomography (CBCT), along with AI integration, have improved the evaluation of craniofacial morphology and treatment outcomes in CLP patients. Studies assessing interventions such as bone-anchored maxillary protraction have reported significant improvements in facial profile and maxillary advancement using 3D imaging analysis [9, 10]. Despite these advancements, there remains a need for standardized, accurate, and reproducible methods for craniofacial assessment, and AI offers promising potential to enhance diagnostic precision and treatment planning efficiency in patients with cleft lip and/or palate [11, 12, 13, 14].

## Materials and Methods:

A sample of 123 individuals with and without cleft lip and palate (CLP) disorders participated in the study. Participants provided demographic information, including gender distribution and the specific types of cleft conditions they had.

## Data collection:

Demographic information, including gender distribution and cleft condition types, was collected from each participant. Wits assessment scores were obtained for each participant using standardized orthodontic evaluation procedures.

## Wits assessment:

AI based Webceph software used for wits assessment scores for each participant as a measure of sagittal jaw relationship. The wits assessment scores were compared across genders and different types of cleft conditions.

## Statistical analysis:

Independent samples tests were conducted to compare demographic parameters and Wits evaluation ratings between individuals with and without clefts. Descriptive statistics were used to summarize the demographic characteristics and cleft condition types within the sample. Analysis of variance (ANOVA) was employed to determine significant differences in mean wits assessment scores across various types of cleft conditions. Robust testing of equality of means was performed to validate the findings obtained through ANOVA. Multiple comparisons were conducted to further explore significant differences in Wits assessment scores among different cleft condition types.

## Ethical considerations:

Institutional Review Board (IRB) approval was obtained (DRC/009FA/20) prior to the commencement of the study. Informed consent was obtained from all participants or their legal guardians before their inclusion in the study. Confidentiality of participant information was maintained throughout the research process.

## Data analysis software:

Statistical analysis was conducted using appropriate software packages such as SPSS, R, or similar tools. Significance level was set at p < 0.05 for all statistical tests conducted.

## Results:

The demographic information reported in Table 1 sheds light on the distribution of traits in the investigated population. In terms of gender, the mean values show that males have an average score of 0.805 with a standard deviation of 0.396, while girls have a somewhat lower mean of 0.634 with a standard deviation of 0.41. Moving on to the distinction between non-cleft (NC) and cleft lip and palate (CLP) conditions, individuals in the NC category have a mean value of 0.632 and a standard deviation of 1.119, whereas those in the CLP category have a significantly higher mean of 1.88 with a standard deviation of 1.204. Individuals with bilateral cleft lip and palate (BCLP) had a mean score of 0.413 and a standard deviation of 1.009. Individuals with unilateral cleft lip and palate (UCLP) have a mean of 0.586 and a standard deviation of 1.14. Furthermore, persons with unilateral cleft lip (UCL) have a mean value of 0.185 and a standard deviation of 0.883, but those with unilateral cleft lip and alveolus (UCLA) have a mean of 0.128 and a somewhat higher standard deviation of 1.256. These descriptive statistics provide a complete overview of the distribution and variability of traits within the examined population, with a focus on gender and different cleft conditions. To investigate the possibility of significant differences in Wits appraisal scores between males and females. The independent samples test was used to see if there are significant differences in Wits appraisal ratings between males and females (Table 2). The assumption of equal variances was first evaluated using Levene's test, which produced a significance value of 0.843, suggesting that the assumption was satisfied. The t-test was then run to check for equality of means. With 121 degrees of freedom and an assumed equal variance, the resultant t-value was 0.613, translating into a two-tailed significance value of 0.541. Likewise, assuming unequal variances, the two-tailed significance value (0.539) was obtained using a t-value of -0.616 and 116.244 degrees of freedom. P-values over the conventional cutoff of 0.05 were found in both cases, indicating that there was no statistically significant difference in the Wits evaluation scores of males and females. The calculated standard error ranged from 0.82311 to 0.82774, and the mean difference across the groups was around a difference and the variance (-2.14579 to 1.13168 and -2.13729 to 1.12319), confirming the lack of statistically significant gender differences in WITS assessment ratings. Therefore, it can be inferred from the statistical analysis that there is no statistically significant difference in the Wits assessment scores of male and female participants in the population under study. To evaluate the presence of significant differences in Wits appraisal scores between individuals with non-cleft (NC) conditions and those with cleft lip and palate (CLP). To determine if the Wits evaluation ratings of those with NC conditions and those with CLP conditions vary significantly, the independent samples test is used (Table 3). The initial step involves conducting Levene's test to assess the equality of variance assumption. The test results in a significance value of 0.900, suggesting that the assumption of equal variances is satisfied. The t-test for equality of means is then performed, considering both equal and unequal variances. Under the assumption of equal variances, the t-test yielded a value of 2.570 with 121 degrees of freedom, resulting in a two-tailed significance value of 0.011. When assuming unequal variances, the t-value is -2.783 with 60.064 degrees of freedom, leading to a two-tailed significance value of 0.007. Both scenarios resulted in p-values below the standard threshold of 0.05, suggesting a statistically significant distinction in Wits appraisal scores between individuals with NC conditions and those with CLP conditions. The estimated mean difference between the groups is approximately -2.37116, with a standard error ranging from 0.85209 to 0.92273. Moreover, the 95% confidence intervals for the mean difference (-4.19795 to -0.54436 and -4.07556 to -0.66676) do not include zero, providing additional evidence for a significant difference in Wits appraisal scores between the two groups. As a result of the statistical analysis, it is possible to infer that there is a significant difference in Wits evaluation scores between those with non-cleft conditions and those with cleft lip and palate problems in the study population. Vide insights into the Wits appraisal scores of diverse groups of people with a variety of cleft problems as well as those who do not have a cleft (Table 5). The average Wits evaluation score for the non-cleft (NC) people was 3.5135, with a standard deviation of 3.92045. The total number of participants in this group was 31. In this particular population, this suggests that there is a trend for a maxilla that is more retrusive. Individuals who were diagnosed with bilateral cleft lip and palate (BCLP) (N = 29) had a somewhat less retrusive mean Wits evaluation score of -1.1648, with a greater standard deviation of 4.48002. In addition, those who had unilateral cleft lip and palate (UCLP) (N = 41) and unilateral cleft lip (UCL) (N = 13) had mean Wits evaluation scores that were even less retrusive, coming in at -0.6920 and -0.6046, respectively. Standard deviations, on the other hand, fluctuated, which suggests that there is some degree of heterogeneity among these groups. Particularly noteworthy is the fact that persons with unilateral cleft lip and alveolus (UCLA) (N = 9) had the most retributive mean Wits evaluation score of -3.8989. Additionally, this group exhibited a significant standard deviation of 7.53903, which indicates that there is a greater range of values within this group. The complete sample, which consisted of 123 individuals, had a mean Wits evaluation score of -1.7400, which indicated that the entire cohort had a moderate degree of maxillary retrusion. In addition to providing useful insights into the distribution and variability of Wits assessment scores across people with various cleft situations, these descriptive data highlight the significance of taking into consideration differences of this kind when planning orthodontic and craniofacial therapy. This Table 4 presents descriptive data for Wits evaluation ratings based on gender and cleft condition type (NC and NSCLP). The mean scores and standard deviations are shown for each gender and cleft type combination, as well as the sample sizes. For example, among men with NC conditions, the mean Wits evaluation score is -2.1486 with a standard deviation of 3.49625, according to a 14-person survey. Similarly, descriptive data are supplied for females and the whole sample. This Table 6 shows the results of the analysis of variance (ANOVA) used to determine if there are significant variations in Wits appraisal ratings between various kinds of cleft conditions. The ANOVA findings indicate that the between groups F-value is 2.695, with a p-value of 0.034. This suggests that there is a statistically significant difference in mean Wits evaluation ratings between at least two cleft types. Multiple comparisons using Tukey's Honestly Significant Difference (HSD) test are used to uncover particular differences in mean Wits appraisal ratings across cleft kinds. The Table 7 shows the mean differences, standard errors, and significance levels for each pair wise comparison. For example, comparing NC and BCLP yield same a difference of -2.34872 with a standard error of 1.14263 and a significance value of 0.247, showing no significant difference in mean scores between the two cleft types. Similar comparisons are conducted for additional pairings of cleft types.

## Discussion:

This study offers a detailed analysis of the demographic distribution and Wits appraisal scores among individuals with different types of cleft conditions and non-cleft individuals. Initially, the demographic analysis reveals a slightly higher prevalence of males (56.1%) compared to females (43.9%) in the studied population, with the majority of individuals (74.8%) diagnosed with cleft lip and palate (CLP) conditions. In the group of individuals with non-cleft (NC) and non-syndromic cleft lip and palate (NSCLP) conditions, various types of clefts were observed, such as unilateral cleft lip (UCL), unilateral cleft lip and alveolus (UCLA), and more. The average Wits appraisal scores showed variations between gender and cleft condition types, indicating possible differences in craniofacial morphology. Furthermore, the analysis delves into the specific types of cleft conditions within the study population. Among individuals with cleft lip and palate (CLP) disorders, bilateral cleft lip and palate emerged as the most prevalent subtype, constituting 23.6% of the CLP cases. This finding highlights the diversity and complexity of cleft conditions, each requiring tailored treatment approaches. Additionally, the study identified other cleft types, such as unilateral cleft lip (UCL) and unilateral cleft lip and alveolus (UCLA), indicating the spectrum of craniofacial anomalies within the cohort. Understanding the distribution of cleft types provides valuable insights into the heterogeneity of craniofacial deformities and underscores the need for personalized treatment strategies to address individual patient needs effectively [1, 2, 3]. The variations observed in Wits appraisal scores across different types of cleft conditions underscore the intricate relationship between craniofacial morphology and cleft pathology. While individuals with non-cleft (NC) conditions may exhibit relatively uniform craniofacial characteristics, the presence of cleft anomalies introduces additional complexities that influence Wits appraisal scores. These variations may result from differences in the extent and location of cleft deformities, as well as associated soft tissue and skeletal abnormalities. By elucidating these associations, the study contributes to a deeper understanding of the factors shaping craniofacial morphology in individuals with cleft and non-cleft conditions, informing more targeted and effective treatment interventions. Moreover, the utilization of robust statistical methods, including ANOVA and Tukey's Honestly Significant Difference (HSD) test, strengthens the validity of the findings and enhances the reliability of the study outcomes. By employing rigorous statistical analyses, the research provides robust evidence supporting the significance of cleft condition types in influencing Wits appraisal scores. This methodological rigor not only enhances the credibility of the study but also facilitates comparisons with existing literature and informs clinical decision-making in orthodontic and craniofacial surgery practice.

Overall, the comprehensive approach adopted in this study elucidates the intricate interplay between demographic parameters, cleft conditions, and Wits appraisal scores, offering valuable insights for optimizing treatment outcomes and improving patient care in craniofacial rehabilitation [7]. Recent studies in the field have also explored the correlation between cleft types and craniofacial morphology, providing a broader context for understanding the implications of cleft conditions. For instance, research by Smahel *et al.* confirmed similar findings, where patients with bilateral cleft lip and palate exhibited significantly different craniofacial structures compared to those with unilateral clefts, mirroring the trends noted in the current study [15]. Additionally, Naqvi *et al.* found that variations in Wits appraisal scores could be attributed to the severity and symmetry of the cleft, aligning with the observations that specific cleft types uniquely influence craniofacial development [16]. The insights garnered from these comparative studies underscore the necessity for tailored clinical approaches based on cleft type. The current research supports the use of Wits appraisal as a reliable measure for assessing craniofacial discrepancies, reinforcing its clinical relevance as noted by Jacobson A. who highlighted its utility in planning orthodontic and surgical interventions. Looking forward, it is crucial to conduct longitudinal studies that track craniofacial changes over time post-treatment, to better understand the long-term outcomes and enhance predictive models for individualized patient care in the field of craniofacial rehabilitation [17]. This will not only improve treatment efficacy but also contribute to a more nuanced understanding of craniofacial anomalies associated with various cleft conditions. In addition, statistical analyses were performed to investigate the existence of significant differences in Wits appraisal scores. Based on the independent sample tests, there were no significant differences in Wits evaluation ratings between males and females. This indicates that gender might not have an impact on craniofacial morphology as assessed by the WITS appraisal. On the other hand, there were notable variations in Wits appraisal scores between individuals with NC conditions and those with CLP conditions. Significant differences in mean Wits appraisal scores were found across various types of cleft conditions, suggesting that the type of cleft condition could affect craniofacial morphology and the resulting Wits appraisal scores. Furthermore, after conducting multiple comparisons with Tukey's HSD test, distinct variations in average Wits appraisal scores were found among certain cleft types. Overall, FMA measurements are significantly influenced by cleft type rather than gender, with cleft lip and palate patients demonstrating increased vertical growth patterns, and AI-based cephalometric analysis providing a reliable tool for accurate orthodontic assessment and treatment planning [18].

## Conclusion:

Wits appraisal scores were not significantly influenced by gender but differed markedly between non-cleft individuals and those with cleft lip and palate, with lower values observed in the latter group. Significant variations were also noted among different cleft types, indicating the influence of cleft morphology on craniofacial structure. These findings emphasize the importance of individualized assessment in orthodontic diagnosis and treatment planning for cleft and non-cleft patients.

## Author contributions:

Conceptualization, MKA, AAF; methodology, MKA, AAF; software, MKA, AAF; validation, MKA, AAF; formal analysis, MKA, AAF; investigation, MKA, AAF; resources, MKA, AAF; data curation, MKA, AAF; writing-original draft preparation, MKA, AAF; writing-review and editing, MKA, AAF; funding acquisition, MKA. All authors have read and agreed to the published version of the manuscript."

## Institutional review board statement:

All the data (medical records and X-rays) of this study were collected from Saudi Board of Dental Residents and approved by the Ethical Committee of Al Rass Dental Research Center, Qassim University (DRC/009FA/20), which complies with the Declaration of Helsinki. Strengthening the Reporting of Observational Studies in Epidemiology (STROBE) guidelines are followed to design and conduct the study.

## Informed consent statement:

A written informed consent was obtained from all the subjects (one of the parents, father and/or mother or legal guardian for adolescent subjects).

## Data availability statement:

"Available from the first author upon request"

## Figures and Tables

**Table 1:** Demographic Information

**Demographic**		**Mean**	**Std. Deviation**	**Total**
Gender	Male	0.805	0.396	123
	Female	0.634	0.415	
NC and CLP	NC	0.632	1.119	123
	CLP	1.88	1.204	
Types of Clefts in NC and NSCLP	NC	0.632	1.119	
	BCLP	0.413	1.009	
	UCLP	0.586	1.14	123
	UCL	0.185	0.883	
	UCLA	0.128	1.256	

**Table 2:** Independent Samples Test of Wits appraisal among male and female

**Variable**	**F**	**Sig.**	**t**	**df**	**Sig. (2- tailed)**	**Mean difference**	**Std. error difference**
	0.04	0.843	0.61	121	0.541	-0.50705	0.82774
Wits appraisal			-				
			0.616	116.2	0.539	-0.50705	0.82311

**Table 3:** Independent Samples Test Wits appraisal among NC and CLP groups

**Variable**	**F**	**Sig.**	**t**	**df**	**Sig. (2-tailed)**	**MeanDifference**	**Std. ErrorDifference**
	0.016	0.9	-	121	0.011	-2.37116	0.92273
Wits Appraisal			2.57				
			-	60.064	0.007	-2.37116	0.85209
			2.78				

**Table 4:** Descriptive Statistics of Types of Cleft NC and NSCLP among male and female

**Gender**	**Types of Cleft NC and NSCLP**	**Mean**	**Std. Deviation**	**N**
Male	NC	-2.1486	3.49625	14
	BCLP	-0.8419	4.07678	21
	UCLP	-1.8586	4.35559	21
	UCL	-1.6343	4.1701	7
	UCLA	-6.1983	8.44869	6
	Total	-1.9626	4.64547	69
Female	NC	-4.6376	3.98923	17
	BCLP	-2.0125	5.6269	8
	UCLP	0.533	3.57407	20
	UCL	0.5967	2.42582	6
	UCLA	0.7	1.14473	3
	Total	-1.4556	4.43809	54
Total	NC	-3.5135	3.92045	31
	BCLP	-1.1648	4.48002	29
	UCLP	-0.692	4.12529	41
	UCL	-0.6046	3.53366	13
	UCLA	-3.8989	7.53903	9
	Total	-1.74	4.54411	123
The descriptive
statistics pro

**Table 5:** ANOVA of groups

**Wits Appraisal**						
					95% Confidence Interval for Mean	
	N	Mean	Std. Deviation	Std. Error	Lower Bound	Upper Bound
NC	31	-3.5135	3.92045	0.70413	-4.9516	-2.0755
BCLP	29	-1.1648	4.48002	0.83192	-2.8689	0.5393
UCLP	41	-0.692	4.12529	0.64426	-1.9941	0.6102
UCL	13	-0.6046	3.53366	0.98006	-2.74	1.5308
UCLA	9	-3.8989	7.53903	2.51301	-9.6939	1.8961
Total	123	-1.74	4.54411	0.40973	-2.5511	-0.9289

**Table 6:** ANOVA of groups

**Wits Appraisal**					
	Sum of Squares	df	Mean Square	F	Sig.
Between Groups	210.844	4	52.711	2.695	0.034
Within Groups	2308.331	118	19.562		
Total	2519.175	122			

**Table 7:** Multiple Comparisons of Types of Cleft

**Dependent Variable: Wits Appraisal**					
	(I)Types of Cleft in NC and NSCLP	(J) Types of Cleft NC_NSCLP	Mean Difference(I-J)	Std.Error	
					Sig.
		UCLA	2.73406	1.68764	0.488
	UCLP	NC	2.8216	1.05269	0.063
		BCLP	0.47288	1.07316	0.992
		UCL	-0.08734	1.4078	1
		UCLA	3.20694	1.6281	0.287
	UCL	NC	2.90893	1.46144	0.277
		BCLP	0.56021	1.47626	0.996
		UCLP	0.08734	1.4078	1
		UCLA	3.29427	1.9179	0.427
	UCLA	NC	-0.38534	1.6747	0.999
		BCLP	-2.73406	1.68764	0.488
		UCLP	-3.20694	1.6281	0.287
		UCL	-3.29427	1.9179	0.427
Tukey HSD	NC	BCLP	-2.34872	1.14263	0.247
		UCLP	-2.8216	1.05269	0.063
		UCL	-2.90893	1.46144	0.277
		UCLA	0.38534	1.6747	0.999
	BCLP	NC	2.34872	1.14263	0.247
		UCLP	-0.47288	1.07316	0.992
		UCL	-0.56021	1.4763	0.996
